# Design, Characterization and Analysis of a 0.35 μm CMOS SPAD

**DOI:** 10.3390/s141222773

**Published:** 2014-12-01

**Authors:** Khalil Jradi, Denis Pellion, Dominique Ginhac

**Affiliations:** Laboratory Electronique, Informatique et Image, Le2i UMR CNRS 6306, Aile de l'ingénieur-9, Avenue Alain Savary-BP 47870, Dijon Cedex 21078; France; E-Mails: denis.pellion@u-bourgogne.fr (D.P.); khalil.jradi @u-bourgogne.fr (K.J.)

**Keywords:** photodetectors, avalanche photodiodes (APDs), optoelectronics, photonic integrated circuit, integrated optoelectronic circuits

## Abstract

Most of the works about single-photon detectors rely on Single Photon Avalanche Diodes (SPADs) designed with dedicated technological processes in order to achieve single-photon sensitivity and excellent timing resolution. Instead, this paper focuses on the implementation of high-performance SPADs detectors manufactured in a standard 0.35-micron opto-CMOS technology provided by AMS. We propose a series of low-noise SPADs designed with a variable pitch from 20 μm down to 5 μm. This opens the further way to the integration of large arrays of optimized SPAD pixels with pitch of a few micrometers in order to provide high-resolution single-photon imagers. We experimentally demonstrate that a 20-micron SPAD appears as the most relevant detector in terms of Signal-to-Noise ratio, enabling emergence of large arrays of SPAD.

## 1. Introduction

Historically, single-photon detection was the domain of photomultiplier tubes (PMT). Since the first demonstration in 1949 [1], PMT devices still play an important role because they offer extremely good performance. However, PMTs have several disadvantages (limited number of pixels, overall dimensions, and high operating voltages), making them unsuitable for compact design of integrated vision systems [2], so the past decade has seen a dramatic increase in interest in new integrated single-photon detectors.

Attractive candidates for the replacement of PMTs are solid-state devices called Single-Photon Avalanche Diodes (SPADs) [3]. SPAD detectors feature both an excellent timing resolution and single-photon sensitivity. SPADs work in avalanche mode above the breakdown level. When an incident photon is captured, a very fast avalanche is triggered, generating an easily detectable current pulse. The first successful CMOS integration of SPAD devices was made less than 10 years ago [4]. Continuous advances provide the potential for integrating large SPAD-based imagers. Recently, remarkable results have been obtained with the implementation of the first SPADs imagers in 130 nm and 90 nm technologies.

However, despite these significant prototypes, there still remain several challenges to be faced for their effective applicability in consumer-applications [5]. Among these challenges, the most relevant one is the use of standard CMOS technology while preserving a level of performance similar to the performance obtained with a customized process. The new generation of SPAD detectors turns towards the integration of large SPAD-based array of pixels with the emergence of several 32 × 32-pixel sensors [6–8] and even some 128 × 128-pixel prototypes [9] in the last years.

Various applications could be envisioned using this kind of photodetectors. For medical applications, the use of SPADs in the field of Positron Emission Tomography (PET) and Magnetic Resonance Imaging (MRI) offers advantages of better spatial resolution, less cost and especially for imaging integration [10]. Also, for fluorescence applications (fluorescence lifetime imaging microscopy and fluorescence spectroscopy) SPADs could be very useful by ensuring better spatial resolution than PMTs provide [11].

In this paper, two principal points will be presented. First, the technological process constituted by the different layers that are used to manufacture the devices. Second, the choice of the optimal photodiode area for the next generation of SPAD Geiger concluded from the results of the electronic and optical characterizations performed on the designed components.

## 2. Structure Design

**Figure 1.**
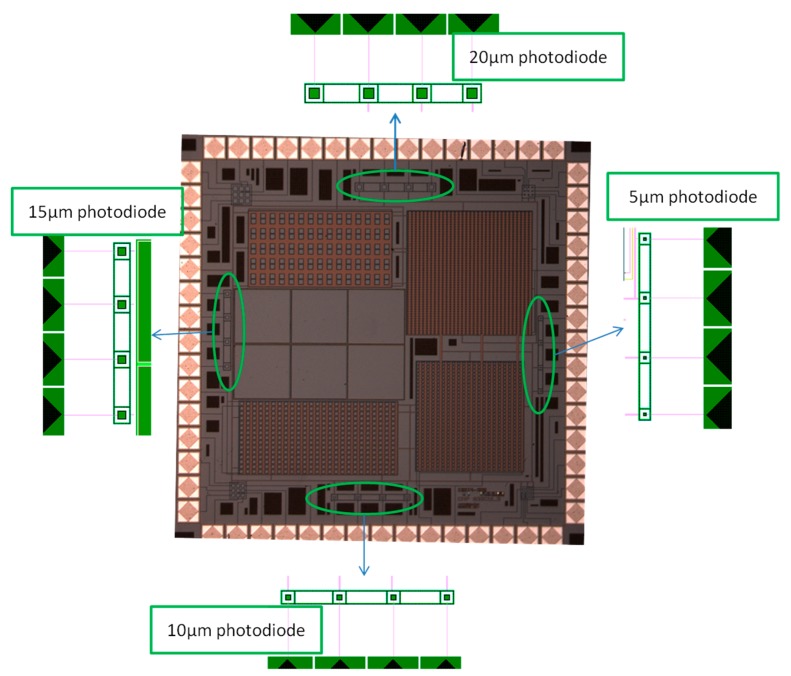
Microphotograph of the chip including zoom on 5 μm, 10 μm, 15 μm, and 20 μm photodiodes.

The AMS 0.35 μm Opto process is an advanced mixed-signal CMOS process, providing four metal layers, two polysilicon layers, high-resistivity polysilicon and two types of transistor gates (3.3 V and 5 V). The process is available through multi-project submission runs, allowing easy and relatively inexpensive prototyping. From a technological point of view, it has also been chosen because it provides an enhanced optical sensitivity for embedded specific photodiodes and capabilities for designing high-density CMOS sensors. Typically, the process allows the design of photodiodes based on N-well/p-epi structures that are optimized for a low dark current at room temperature. The photodetector structure is based on a p^+^ substrate to which an epi-layer is linked through p^−^ type of 14 μm thickness and doping of approximately 2 × 10^17^ cm^−3^. Both layers constitute the initial substrate. Many processes are accomplished to reach the n/p junction. The corresponding technological process is shown in Figure 1.

**Figure 2.**
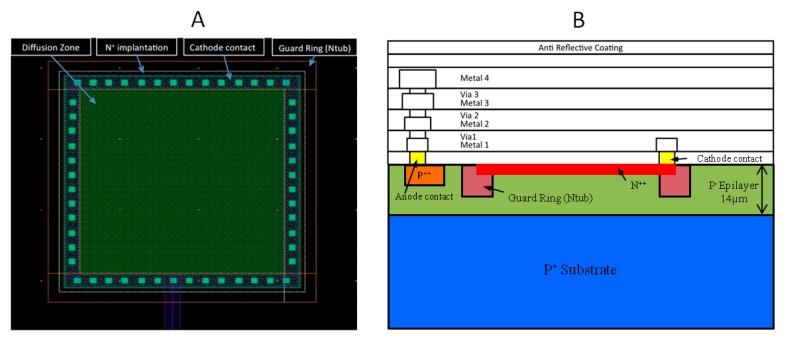
(**A**) Photodiode layout; (**B**) Cross section of the CMOS SPAD.

Figure 2 shows the layout and the cross-section of the photodiode implemented in the AMS 0.35 μm Opto process. Note that in designing such a device, only standard masks provided by the technology are used. The photodiode depicted in Figure 2A is an n/p junction with a thin n layer (about 0.2 μm thick of arsenic) deposited on the p epi-layer (about 14 μm thick of boron); the n layer corresponds to a doping of approximately 1 × 10^20^ at·cm^−3^. A strong and uniform electric field within the depletion region allows for the avalanche formation and the single photon sensitivity of the photodiode. This field must be distributed as uniformly as possible at the center of the junction. Moreover, the photodiode periphery must be protected to prevent premature breakdown. To avoid this detrimental effect, the photodiode must be surrounded by a suitable guard ring (GR). Two main families of guard rings designs are available from literature: for 0.35 μm CMOS processes and above, guard rings generally consist of a lesser doped region (n-well structures or p-well implants) surrounding the active part of the photodiode and thus lowers the electric field at the borders of the diode to avoid premature edge breakdown [4,12]. For modern technologies (beyond 250 nm), the use of Shallow Trench Isolation (STI) as the guard rings was introduced and proposed as the most promising alternate to conventional guard rings [13,14]. In our designs, the guard ring structure is based on an explicit low-doped implanted n-well region (of about 3.5 μm thick phosphorus) that surrounds the periphery of the multiplication region of the photodiode as shown in Figure 2B. In this case, the corresponding breakdown voltage of the volumetric region (active area/epi-layer junction) is estimated to be 12 V depending on photodiode size and wafer homogeneity. Breakdown voltage of GR/epi-layer junction is estimated to be 35 V.

The manufactured chip includes several types of devices such as a significant number of elementary pixels with different sizes (5, 10, 15 and 20 μm side of active area of square photodiode), SiPM (matrix of pixels associated in parallel), 3 × 3-pixel imager and also test zones. This paper focuses only on the characterization of the elementary photodiodes in order to provide some new results about the impact of the size of photodiodes on single photon counting.

## 3. Characterization Results

Two series of characterizations have been conducted for each size of photodiode: a static characterization and a dynamic characterization including surface, noise and light studies. The aim of these studies is to focus on the selection of an optimal size to be further integrated in imaging systems.

**Figure 3.**
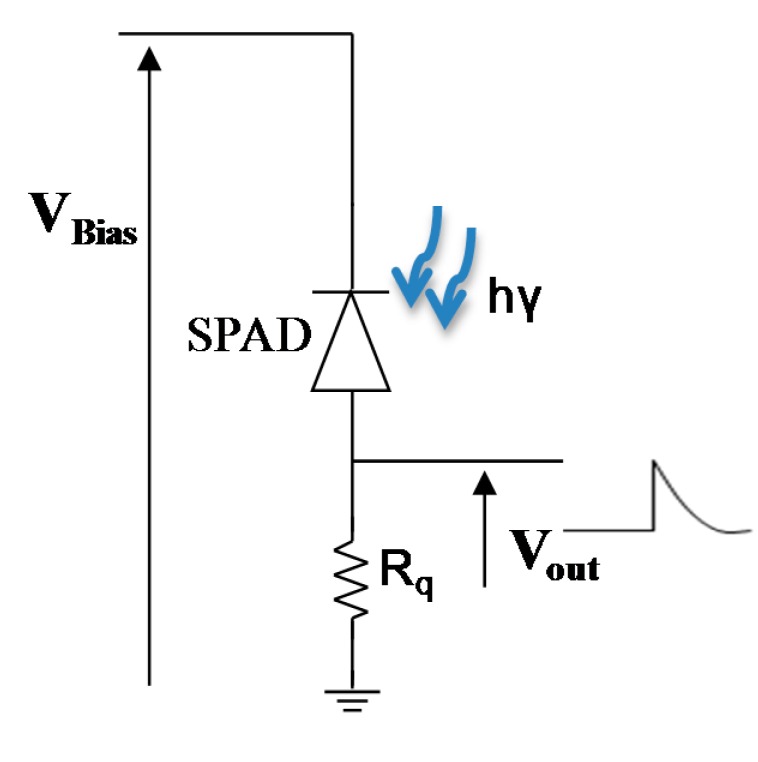
Electrical circuit used for both static and dynamic (dark and light) characterizations, an exterior quenching resistor has been used.

The electrical circuit used for these characterizations is presented on Figure 3.

### 3.1. Static Characterization

We start characterizing the SPADs with the evaluation of the static current-voltage characteristic in the reverse bias mode of operation. The following experiment is performed on each SPAD: a Keithley 2636 A source meter, connected to a computer, obtains measurements of current in reverse mode. This experiment is achieved by generating a sweep voltage between 0 and 14 V then measuring current and limiting it to 20 μA (to avoid damage to the photodiodes). The current *versus* voltage characteristic reveals two important parameters of photodiodes in Geiger mode: the breakdown voltage *V*_Br_ and the dark current *I*_d_. Table 1 recapitulates the breakdown voltage and the dark current according to each photodiode size.

Note that, 5 μm photodiode size presents particular features compared to other photodiodes of larger sizes. This behavior is related to the phenomena of guard rings lateral diffusion. This diffusion represents an important part in the photodiode central region (which is originally very small). Consequently, the breakdown voltage is dominated by the peripheral breakdown, leading to a high breakdown voltage [14].

**Figure 4.**
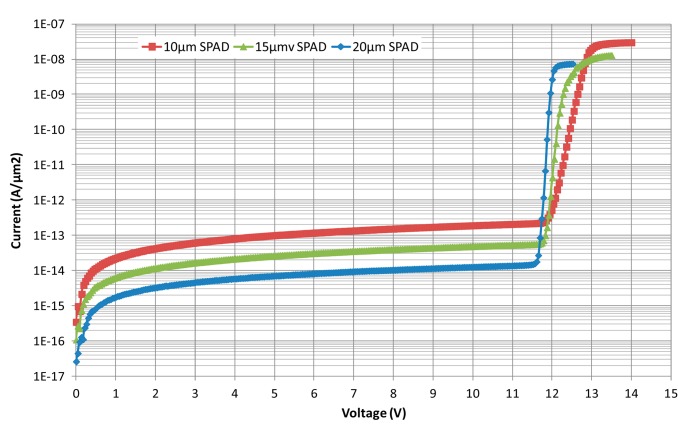
Current *versus* voltage as a function of area for 3 photodiodes (10, 15 and 20 μm).

Figure 4 shows measured *I-V* characteristics of the different SPADs per area unit (A/cm^2^) in reverse mode. As previously mentioned, the 5 μm photodiode has been deliberately rejected because of its high breakdown voltage and its high dark current in comparison to its surface. Therefore, Figure 4 focuses only on typical 10, 15, and 20 μm devices. They exhibit abrupt breakdown around 12 V, at 12 V, 11.8 V, and 11.7 V, respectively. It shows that the breakdown voltage tends to reduce with increasing the device size. Such results are similar to those obtained with a 130 nm technology [15].

### 3.2. Dynamic Characterization

Based on the static characterization results, a full dynamic characterization of the SPADs has been performed, as reported in the following paragraphs. All measurements refer to typical 10 μm, 15 μm and 20 μm SPADs, involving for each photodiode different overvoltages, *i.e.*, the difference between the bias voltage and its specific breakdown voltage. A first series of measurements has been conducted to evaluate the Dark Count Rate (DCR) of the SPADs. In a second step, we evaluate the capabilities of the designed SPADs to efficiently count the photons emitted by a light source.

#### 3.2.1. Noise Study

The DCR is measured by counting the number of avalanches while the SPAD is operating at an excess bias voltage above the SPAD's breakdown voltage. The DCR measurement is carried out in the dark condition where the chip is shielded completely not to receive any light. In such a dark environment, a specific electronic card is used for processing pulses delivered by each photodiode. The DCR for the different SPADs has been measured at different overvoltages at room temperature. Overvoltages from 0 V to 1.1 V with a 0.1 V step are applied to the breakdown voltage of each SPAD.

**Figure 5.**
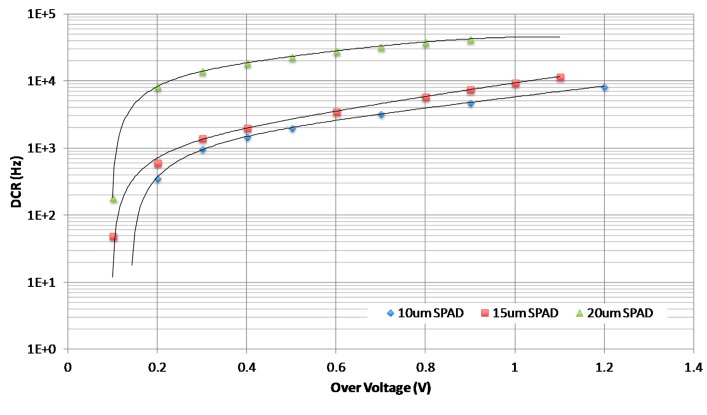
DCR as a function of overvoltage.

Figure 5 shows the raw DCR of the three different fabricated SPADs as a function of the overvoltage. Passive quenching is being used in the implemented SPADs. DCR increases with overvoltage and with photodiode size. DCR vary from 0.3 kHz (10 μm) to 8 kHz (20 μm) at 0.2 V excess bias and from 7 kHz (10 μm) to 13 kHz (20 μm) at 1.1 V excess bias, respectively. The 20 μm photodiode presents the highest DCR whereas the two other photodiodes (10 μm and 15 μm) have both very close DCR.

Our measures are in line with previous results obtained on similar SPAD structures designed in a standard 0.35 μm technology. For example, Niclass *et al.* [16] highlighted an average DCR of 750 Hz for a photodiode with an active area diameter of 10 μm. Similarly, Stoppa *et al.* [17] characterized a series of 17 SPADs with a 20 μm × 20 μm size, showing a mean DCR around 4 kHz. Tisa *et al.* [18] proposed a compact 50 μm × 100 μm cell integrating a 20 μm SPAD. DCR is in the range (2 kHz–5 kHz) for different values of excess bias. Nissinen *et al.* [19] evaluated the dark count rates of different SPADs (deep-n-well cathodes of 10 μm and 20 μm and n-well cathode of diameter 20 μm) as a function of the excess bias voltage at room temperature. The DCR of the 20 μm SPAD with the deep-n-well cathode is approximately 11 kHz, when an excess bias voltage of 3.3 V is used, but because of the higher doping concentration of the n-well, the DCR of the SPAD with the n-well cathode is more than a couple of decades larger than this. The DCR of the 10 μm SPAD varies from 200 Hz at an excess bias of 1 V and converges to 1 kHz for higher overvoltages. We also found in literature some studies with larger structures. Such SPADs are characterized by very high values of DCR. As examples, Stoppa *et al.* [17] characterized an array of 140 μm × 70 μm SPADs, highlighting a very large value of 10^6^ Hz at room temperature. Similarly, Vilella *et al.* [20] presents a 10 × 43 SPAD detector of 20 μm × 100 μm. The mean DCR of the chip is approximately 67 kHz (median value is 40 kHz) with an excess bias of 1 V and 139 kHz with an excess bias of 2 V (median value is 95.3 kHz).

#### 3.2.2. Light Study

**Figure 6.**
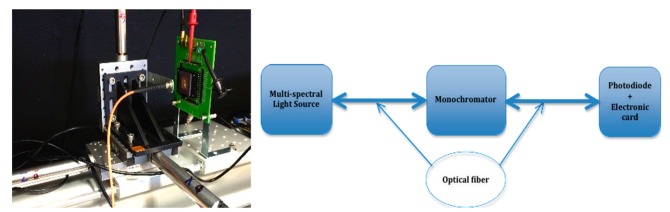
The test bench used for photodiode characterization in light.

The sensitivity of the SPADs structures has been measured over the visible range at different overvoltages, by means of a dedicated test bench including a multi-spectral light source (B&W Tek BPS120-CC), a monochromator (Spectral Products CM110) for the selection of precisely one wavelength, and an optical power meter (Newport 1830-R with 918D-SL-03D Silicon Detector) as shown in Figure 6.

Traditionally, measures of SPADs sensitivity require an integration sphere with a reference diode at one port of the sphere and the SPAD at the other. Unfortunately, our test bench does not include an integration sphere and we evaluate sensitivity by means of the following protocol. The test bench ends with an optical fiber fixed on a point-to-point motion, allowing to precisely align the optical fiber with the chip to evaluate. The alignment has been done in order that the size of the light spot covers the sensitive area of the SPAD as precisely as possible. This can be done by computing the numerical aperture (NA) (0.22 in our case) of the optical fiber and the distance between the head of the optical fiber and the chip, as following:

(1) NA.=n*sinθ

where θ is the half-angle of the maximum cone of light and n is the index of refraction (*i.e.*, *n* = 1 for air). The spot diameter *D*_S_ can be written as:

(2) $$D_{s}=2*D_{fc}*\tan\theta$$

where *D*_fc_ is the fiber-to-chip distance, θ equal 12.7° according to Equation (1).

For *D*_fc_ ≈ 1.3 mm, therefore *D*_S_ = 0.58 mm according to Equation (2).

Hence, the spot area A_S_ can be calculating as following:

(3) $$A_{s}=\pi *\frac{{D_{s}}^{2}}{4}$$

The obtained value is *A*_S_ = 0.27 mm^2^. Knowing the area of each photodiode *A*_P_ (0.0001 mm^2^, 0.000225 mm^2^ and 0.0004 mm^2^ for 10 μm, 15 μm and 20 μm respectively), the ratio area *R*_A_ between the photodiode and the spot has been respectively determined as 2700, 1200, and 675 for the three photodiodes.

**Figure 7.**
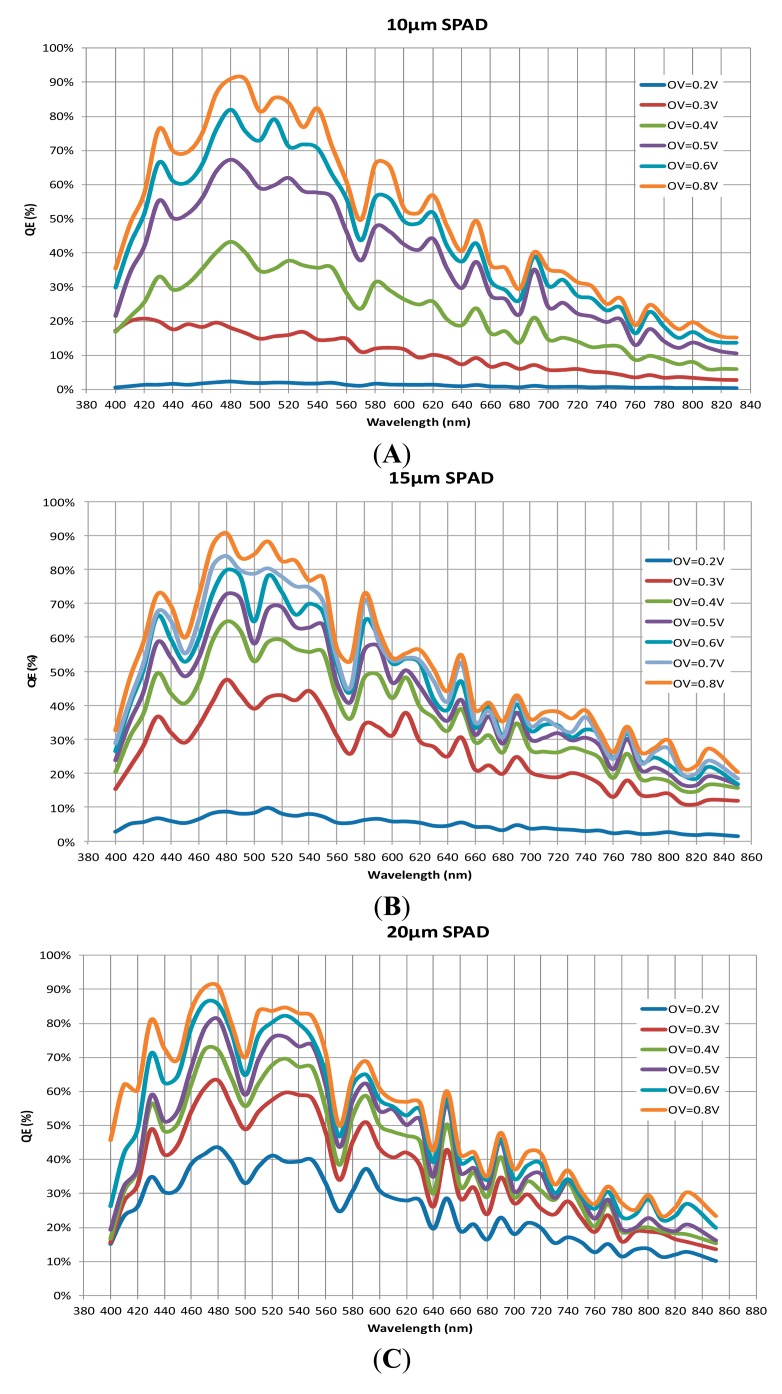
Quantum efficiency of the three SPADs as a function of wavelengths and overvoltages. (**A**) 10 μm SPAD; (**B**) 15 μm SPAD and (**C**) 20 μm SPAD.

Using such a protocol, we are able to precisely compare quantum efficiency (QE) of each size of photodiodes over wavelengths in the range (400 nm, 820 nm) and overvoltages in the range (0, 1 V), as depicted in Figure 7. The three different photodiodes achieve peaks in the blue region (around 480 nm) of the visible wavelength range. This value is comparable with previous results obtained with the same 0.35 μm technology [16,18,21]. The efficiency remains high over a large range of wavelengths, above 50% between 410 nm and 640 nm for overvoltages higher than 0.4 V. Note that, for all the subsequent results described in this paper, the wavelength has been fixed to 480 nm.

**Figure 8.**
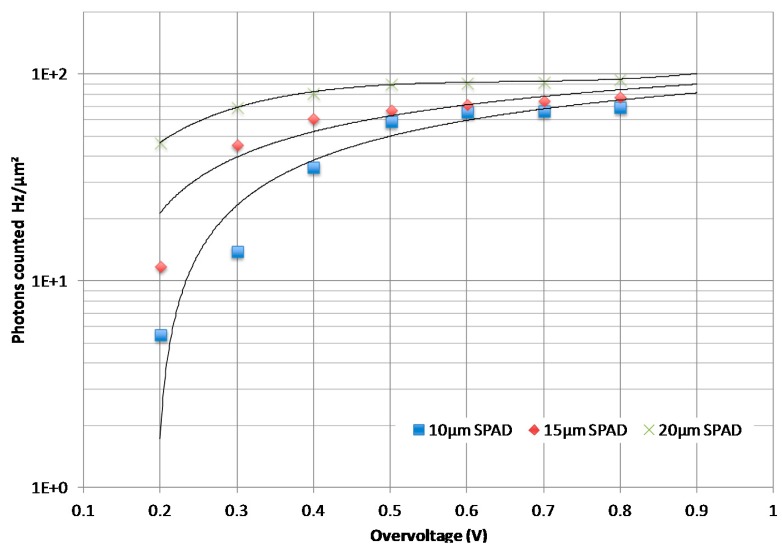
Number of photons counted per area unit as a function of overvoltage.

Since the number of photons that can be detected by a photodetector is a function of its area, it is relevant to focus on the number of photons counted per area unit (counts/mm^2^). This allows a pertinent comparison of the performance of each SPAD. As depicted in Figure 8, the number of photons counted per area unit increases with the size of the photodiode for low values of overvoltage. As an illustration, it is respectively equal to 5.5 × 10^8^, 1.2 × 10^9^, and 4.7 × 10^9^ for the 10 μm, 15 μm, and the 20 μm photodiode for an overvoltage equal to 0.2 V. This is mainly due to the capacity of the large photodiodes (20 μm) to collect a maximum of incident photons comparing to smaller photodiodes (10 μm and 15 μm). The number of photons counted also increases with overvoltage until 0.7 V–0.8 V, where saturation occurs.

**Figure 9.**
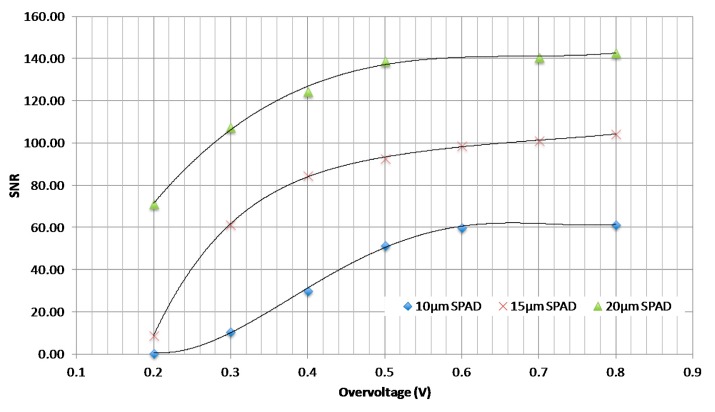
SNR as a function of overvoltage.

We also evaluated the Signal to Noise Rate (SNR) for each photodiode as a function of overvoltage [22], as seen on Figure 9. The SNR is calculated using the following equation:

(4) $$\text{SNR}=\frac{\text{Signal}*QE*T_{i}}{\sqrt{\text{Signal}*QE*T_{i}+\text{Noise}*T_{i}}}$$

where, the Noise is the DCR of each photodiode that is already measured (cf. Figure 5). *T*_i_ is the time integration (equal 1 s in our measure). The Signal is the difference between the maximum of photons counted (using the frequency counter) and the noise of each photodiode.

According to these results, the 20 μm photodiode presents the maximum of SNR compared to other photodiodes (SNR = 140 at overvoltage > 0.5 V), and represents the best compromise in terms of area and performance. This result is similar with the 20 μm pixel designed by Stoppa *et al.* [17] exhibiting an average dark count rate of around 4 kHz and a dynamic range of over 120-dB. It is also comparable with the 10 × 10-SPAD detector module [23] including a pixel pitch of 26 μm. Each of the 100 SPADs has a mean DCR lower than 2 kHz and a dynamic range exceeding 120 dB. Consequently, based on the literature and the deep characterization of our chip, we can conclude that the 20 μm photodiode appears as the optimal candidate for a further integration into a two-dimensional SPAD imager designed with the AMS 0.35 μm Opto process.

## 4. Conclusions

We have presented a detailed investigation concerning Single Photon Avalanche Diodes designed with the AMS 0.35 μm Opto process. Focus has been placed on the photodiode size in order to quantify as precisely as possible the influence of the size on performance, both in terms of DCR and detection. Each of the three devices that have been evaluated features single-photon counting capabilities, enabling further integration into high-resolution arrays. However, one of these structures based on a 20 μm photodiode achieves improved performance, both in terms of quantum efficiency and SNR. Then, this photodiode becomes the best candidate for integration into the next generation of Geiger APD photodetectors including SIPM-based detectors and SPAD-based imagers.

## Figures and Tables

**Table 1.** Breakdown voltage and dark current for each photodiode size.

**Size**	**5 μm**	**10 μm**	**15 μm**	**20 μm**
Breakdown voltage	21.25 V	12 V	11.8 V	11.7 V
Dark current	200 pA	60 pA	85 pA	106 pA
